# Application of Artificial Intelligence in Diabetes Education and Management: Present Status and Promising Prospect

**DOI:** 10.3389/fpubh.2020.00173

**Published:** 2020-05-29

**Authors:** Juan Li, Jin Huang, Lanbo Zheng, Xia Li

**Affiliations:** ^1^Clinical Nursing Teaching and Research Section, The Second Xiangya Hospital, Changsha, China; ^2^Department of Metabolism and Endocrinology, The Second Xiangya Hospital, Central South University, Changsha, China; ^3^National Clinical Research Center for Metabolic Diseases, Changsha, China; ^4^School of Logistics Engineering, Wuhan University of Technology, Wuhan, China

**Keywords:** artificial intelligence, diabetes, diabetes education, diabetes management, AI applications

## Abstract

Despite the rapid development of science and technology in healthcare, diabetes remains an incurable lifelong illness. Diabetes education aiming to improve the self-management skills is an essential way to help patients enhance their metabolic control and quality of life. Artificial intelligence (AI) technologies have made significant progress in transforming available genetic data and clinical information into valuable knowledge. The application of AI tech in disease education would be extremely beneficial considering their advantages in promoting individualization and full-course education intervention according to the unique pictures of different individuals. This paper reviews and discusses the most recent applications of AI techniques to various aspects of diabetes education. With the information and evidence collected, this review attempts to provide insight and guidance for the development of prospective, data-driven decision support platforms for diabetes management, with a focus on individualized patient management and lifelong educational interventions.

## Introduction

Diabetes mellitus (DM) is a long-term chronic disease. According to recently published data from the International Diabetes Federation, the number of people worldwide suffering from diabetes has reached 451 million (1). After undergoing a long period of fast economic growth and corresponding lifestyle changes, China is now facing many issues, such as demographic aging, urbanization, and the largest number of diabetes patients (~114.4 million) in the world (2). Characterized by an insidious onset, numerous complications, and high rates of death and disability, diabetes is an immense burden on individuals and society. Therefore, the prevention and management of diabetes is extremely important and diabetes education is an essential component. Over the past several years, increasing AI-based tools have been developed for diabetes healthcare (3). Patients are supported with more flexible and scholarly access to skills and knowledge for various aspects of diabetes self-management, including diabetes prevention, lifestyle and dietary guidance, exercise, insulin injection, and complications monitoring, to list a few. This paper reviews these advances.

## The Concept of AI and Its Advantages

### The Concept of AI

Artificial intelligence is an interdisciplinary concept based on the foundations of computer science, control theory, information theory, neuropsychology, philosophy, and linguistics (4). In general, AI employs artificial methods to impart intelligence on computers. The term “artificial intelligence” was proposed as early as 1956. In the studies that followed, researchers expanded the concept and proposed nutritious theories and technologies to enable human intelligence by computers. It is believed that by mimicking and improving on the functions of the human brain, AI can free humans from tedious mental and physical work.

Artificial intelligence, sometimes called machine intelligence, is a broad topic. Depending on the objectives of the diabetes-related application, AI methodologies can be classified into three categories: exploring and discovering information, learning to use information, and extracting conclusions from information (3). Searching and designing algorithms to find potential information from databases is commonly known as “knowledge discovery in databases” (KDD). The main goal of KDD, which requires both a broad and a deep knowledge of the area of study of interest, is to identify useful and understandable information. The most representative technologies used in this type of research are the K-means, K-nearest neighbors (KNN) algorithms and hierarchical clustering. As for the learning from knowledge category, the idea is to enable a machine to learn automatically without human intervention or assistance, so that it is able to make predictions on the future status of complex systems for better decision making. This process includes all methods that involve inductive components and they are adaptively used in different cases with their own strengths. The most commonly used techniques are artificial neural networks (ANNs), support vector machines (SVMs), random forest (RF) algorithms, evolutionary algorithms (EAs), deep learning (DL), naive Bayes (NB), decision trees (DTs), and regression algorithms (RAs). The final category, extracting conclusions from information, is often conducted through expert systems. Those systems generally contain three main components: knowledge acquisition interfaces, knowledge bases of rules and information, and inference engines. Those systems process ambiguous concepts and uncertainty via information learned from previous cases and expert knowledge and provide decision-making support for new cases. The most commonly used techniques in this field include rule-based reasoning (RBR), case-based reasoning (CBR), and fuzzy logic (FL).

### AI Advantages in Medical Science

The application of AI in medical science is becoming comprehensive and has yielded immense benefits. The key advantages include broad coverage, low cost, high efficiency, portability, diversity, and service-end “productivity.” In addition, AI enhances the path toward full-course, individualized, and intelligent chronic disease management. Some of the most representative and active results are from medical expert systems. Through the use of AI technologies, the expert systems can make inferences and judgments based on large bodies of professional knowledge and experience that have been accumulated by medical experts, thereby emulating the decision-making processes used by human experts (5).

AI technologies, such as machine learning and data mining, have been increasingly integrated with medical science and are gradually becoming important drivers of medical development. AI presents unique advantages in disease diagnosis and prediction, surgical robotics, image recognition, virtual medical assistance, pharmaceutical research and development, and health management (6–11). In the field of diabetes education, AI technologies have been extensively utilized in diabetes prediction, dietary and exercise guidance, insulin injection guidance, monitoring of complications, and self-management.

## Current State of Diabetes Education/Management and Existing Problems

It is well-accepted that self-management is essential to reduce the risks of chronic complications in diabetes patients. Diabetes education is a system-wide and individualized way to ensure the effectiveness of patient self-management (12). Considering the variety of individual needs, goals, and life experiences, patients should be educated with fine-tuned knowledge and skills that fit their own situation. This kind of patient-oriented education has been proven to improve clinical outcomes and reduce management costs (13). The treatment guidelines used in the United States, Britain, and China all specify that a team of professionals with different areas of specialization should be established to provide education on diabetes management to patients and their family members, thereby ensuring that patients possess effective self-management skills (14–16).

The importance of diabetes self-management education was recognized in the early 1970s to 1980s (17). However, despite this recognition, most of the evidence relevant to this topic was obtained from before-and-after comparisons of patients with uncontrolled diabetes or from non-randomized control studies (18, 19). Diabetes education helps patients to develop self-management skills to manage their own incorrect behaviors, and therefore, various methods are used to customize patient-oriented programs. While patients are the greatest beneficiaries of their educational intervention, a recent study that covered 12 quantitative and qualitative methods revealed that a relatively large percentage of patients refuse to participate in educational programs. The reasons include lack of time, excessive class time, class contents that are not suitable for the patient, and unaffordable travel expenses (20). In addition, one offline diabetes education program can only serve 10–20 patients at a time, while there is a worldwide shortage of diabetes specialists, nurses, and diabetes education personnel (21). This suggests that new methods and models of educational intervention should be explored to improve efficiency and coverage such that the long-term and patient-specific requirements can be satisfied.

In recent years, diversified models for diabetes education programs have been designed and conducted, including diabetes management applications (apps), summer camp activities, interventions by interdisciplinary teams, remote interventions, sequential health education, peer education, large-class education, and small group education. Although AI platforms based on apps and web services are becoming an effective alternative that is able to provide education to a massive number of diabetes patients, the percentage of physicians and patients willing to use this technology in diabetes management remains relatively low (22). One of the major flaws is that the functions do not satisfy patients' needs. The underlying reason is that the apps were designed without the participation of medical professionals. Without incorporating recommendations from physicians on diagnosis or treatment, the apps lack the high-quality proof of effectiveness that can be obtained from clinical trials (23).

The task of designing and implementing diabetes educational systems is very complicated and requires that interdisciplinary experts work together to obtain the best outcome. Although various educational methods with more or less pros and cons exist, problems such as how to use existing technologies to optimize educated decisions, how to provide patient-specific guidance and individualized management plans, and how to conduct long-term, effective educational intervention for lifelong chronic diseases are the focus of what needs to be addressed by diabetes education.

## Current Status of AI Applications in Diabetes Education and Management

Compared with the conventional education methods, AI-based education offers significant advantages including low costs, easy implementation, broad coverage, flexible doctor-patient interaction, avoidance of repeated efforts, reduction in the workload of medical personnel, and an enhancement in effectiveness. Current AI technologies applied to diabetes education mainly focus on diabetes prediction, lifestyle guidance, insulin injection guidance, blood sugar monitoring, self-management, and complication monitoring. Here, we try to illustrate as comprehensive as possible the existing AI methods that have been applied in diabetes management from various aspects.

### Diabetes Prediction

To predict the risk of adults developing type 2 diabetes, British scholars performed a systematic assessment of 94 existing type 2 diabetes risk assessment models and scoring standards using both quantitative and qualitative methods. They also selected some of these models and scoring methods and applied them in practice. The experimental results proved the stability of a considerable proportion of the risk assessment approaches and verified these approaches in different patient groups (24). A study by Mani et al. (25) used various machine learning algorithms in conjunction with electronic medical record data from 2,000 patients with type 2 diabetes to predict the risk of diabetes. It was found that the area under the curve (AUC) values of 6-months and 1-year predictions were <0.8. This indicated the feasibility of using electronic medical record data to automatically predict diabetes and identify persons at a high risk of diabetes. Fu et al. developed a risk assessment model for postprandial hyperglycemia that can be used to identify persons at a high risk for this condition to ultimately reduce the number of people who must take an oral glucose tolerance test (OGTT) (26). In this sense, we hold this thought that different assessment models should be developed for different races and AI models focusing on postprandial hyperglycemia are extremely promising to improve the efficiency of diabetes prediction.

### Lifestyle Guidance for Diabetes Patients

Understanding the carbohydrate content of individual diets and how carbohydrates affect blood glucose is one of the cornerstones of diabetes treatment. To appropriately adjust an insulin dose, the total amount of carbohydrates ingested by the patient must be calculated. As early as the 1990s, some scholars used a computerized device (a food meter) to help 21 diabetes patients record the types and amounts of food and beverages they consumed during a 1-week period (27). This approach shed light on investigating dietary types and characteristics of patients so as to provide individualized insulin dose selection advice for better blood glucose management. An experimental study indicated that compared with relying solely on carbohydrate levels to perform insulin dose calculation, taking fat and protein into consideration may lead to a significant reduction in hemoglobin A1C (HbA1c) levels (28). Software applications that use graphic analysis technologies to quickly and directly analyze dietary content are under development. The proposed software allows patients to quickly obtain relevant nutrition information, including carbohydrate content and calories, by taking smartphone photos and will thereby help in their nutritional choices (29). Recently, a study by Oka et al. (30), employed a deep learning and remote communications technology to analyze photographs of patients' meals and thereby automatically assess nutrient intake. The results of a 12-months randomized controlled experiment showed that the average variation in HbA1c level was 0.3% with a standard deviation of 0.3%. This indicated that nutritional intervention through automated AI support yields results similar to those obtained with face-to-face nutritional intervention support from a human dietitian. These methods can therefore greatly reduce the load on the workforce of human experts, while simultaneously enhancing the effectiveness of dietary guidance for diabetics.

Zeevi et al. (31) designed a machine learning algorithm that was able to integrate the blood parameters, dietary habits, blood measurements, and physical activities of an 800-person cohort and verified that the algorithm was able to accurately predict postprandial glycemic responses to real-life meals for the individuals in a 100-person cohort. The results of this experiment indicated that individualized diets can successfully reduce postprandial glucose elevation and its metabolic consequences.

Proper exercise plays an important role in blood sugar control and reduce the risk of cardiovascular events. The Action for Health in Diabetes (Look AHEAD) trial relied on machine learning to reanalyze data and confirmed that type 2 diabetes patients can benefit from intensive lifestyle intervention involving weight loss, which can also reduce the incidence of cardiovascular events (32). During the early stage of diabetes, commitment to a healthy diet and weight loss can be employed as preventive measures. Everett et al. (33) proposed a decision support system using machine learning and found that this system was able to promote compliance with physical activity and weight loss goals of the users and therefore reduce their risk of developing diabetes. Jacobs et al. (34) proposed a regression model that could be used to automatically analyze the exercise levels of patients wearing accelerometers and heart monitors and monitor changes in glucose levels that occurred while the subjects were exercising. The results from an assessment of 13 type 1 diabetes patients had a sensitivity of 97.2% and a specificity of 99.5%.

With the technical progress of picture visualization and the widespread use of wearable devices, we believe that AI would play a crucial role in personalized lifestyle guidance and thus help the management of diabetes.

### Insulin Injection Guidance

For patients with type 1 diabetes, lifelong dependence of insulin is the only effective treatment (35). Published data from the American Diabetes Control and Complication Trial (DCCT) indicated that a 1.9% decrease in HbA1c through early intensive insulin treatment could significantly decrease the risk of complications and death (36, 37). Furthermore, the most recent evidence indicated that large doses of insulin may be associated with adverse cardiovascular outcomes in patients (38). Insulin is therefore a double-edged sword, and should be used in a safe and effective manner.

In theory, insulin dosage can be calculated using formulas with the following inputs: carbohydrate coefficient, insulin sensitivity coefficient, and data collected from dynamic blood sugar monitoring; however, AI technologies have been intensively applied to this aspect of diabetes management to provide better insulin usage support for patients. In a recent multicenter, randomized controlled study, Bergenstal et al. (39) used the d-Nav insulin guidance system to monitor and predict glucose levels so as to automatically advise patients appropriately on their next insulin dose. The data used by the researchers included glucose readings (sensor readings or fingertip blood glucose measurements), insulin dose records, dietary carbohydrate intake, and other relevant information. The algorithmic core was an event-driven machine learning method. The results indicated that HbA1c decreased by an average of 1.0% from baseline levels to 6 months in the intervention group and the difference compared to the control group was significant. These experimental results indicated a potential use of the d-Nav insulin guidance system for stable blood sugar control and the optimization of insulin management in type 2 diabetes patients.

In another study, Nimri et al. (40) worked on a closed-loop remote monitoring system called MD-Logic. The analysis is based on fuzzy logic and a self-adaptive learning algorithm. A randomized controlled experiment involving 75 diabetes patients was performed to observe the occurrence of nocturnal hypoglycemia. This study found that the system could significantly reduce the median duration of nocturnal hypoglycemia (*P* = 0.004). Considering its effectiveness at reducing the risk of nocturnal hypoglycemia, the authors concluded that the MD-Logic system can be used safely at night by children, adolescents, and type 1 diabetes patients.

Logic-based AI tools are also utilized to provide insulin guidance. Researchers have tried to determine the effects of eating time, drinking time, and exercise time on individual glucose metabolism via case-based reasoning (CBR). This method was used to calculate an individualized insulin bolus using an insulin intravenous bolus calculator, thereby achieving optimal glucose levels in patients and optimizing insulin treatment (41).

Most recently, it was reported that an app named CamAPS FX has become the world's first available artificial pancreas app to people with type 1 diabetes in UK (42). This is a huge step for the AI application in the automomic calculation of insulin dosage and would tremendously improve the metabolic control of insulin-treated patients with diabetes.

### Blood Sugar Monitoring

Numerous studies have verified that continuous glucose monitoring (CGM) employing an intelligent algorithm combined with insulin pump therapy can help patients achieve a better understanding of their blood sugar fluctuations. Compared with traditional monitoring methods, CGMs provide the whole picture of blood sugar and can better detect occult hypoglycemia, making it an excellent means of observing blood sugar fluctuations and enhancing self-management ability (43, 44). A recent study used a neural network system to perform a comparative assessment of four blood sugar prediction models used by type 1 diabetes patients. The researchers used data from 10 type 1 diabetes patients, including data on physical activity obtained by means of sensors, and found that the model was able to effectively predict the risk of hypoglycemia (45). In the United States, the WellDoc diabetes management system, a smartphone software application, has been approved by the FDA and is covered by most types of medical insurance. Doctors can use this system in conjunction with patients' blood sugar diaries to provide individualized feedback and recommendations via real-time online communication. Doctors can also prescribe the app to patients so that they can continue to record their blood sugar levels in real time and upload dietary and drug treatment information. Quinn et al. (46) used this app to perform a randomized control trial involving type 2 diabetes patients and found that patients using the smartphone app to manage their diabetes had A1c levels that were 1.2% lower than those of the control group who received drug treatment alone. In China, Ling et al. (47) assessed the effectiveness of the mobile health app TangTangQuan (TTQ) in the management of type 1 diabetes; patients used the TTQ system to record and upload their diabetes diaries. The patients and their companions or family members were able to provide their comments, share their own experiences, and interact with researchers. The results of this study indicated that TTQ can be used as a means of intervention to promote active communication with type 1 diabetes patients and family members and can help support diabetes self-management education.

Thanks to the rapid development of the Internet and AI technologies, decision support systems (DSSs) are already being used in the management of diabetes treatment. These systems can record information on diet, exercise, pharmaceutical use, and blood sugar levels and can be combined with patient- and physician-support tools for the purpose of improving disease outcomes (48, 49). The use of AI technology and online applications in diabetes management is becoming increasingly effective in both the United States and China (48, 49).

### Monitoring of Diabetes Complications

The most common complications of diabetes include vascular pathologies and peripheral neuropathies. Scholars who assessed adult retinal fundus photographs found that detection of diabetic retinopathies by a deep learning algorithm achieved sensitivity and specificity of more than 93% (50). In a retrospective analysis of 9,939 posterior pole photographs from 2,740 diabetes patients, Takahashi et al. (51) confirmed that deep learning can be used to assess diabetic retinopathies. Based on their results, Takahashi et al. presented a new AI disease grading system that can be used to grade the severity of diabetic retinopathies.

Likewise, a mobile app called “FootSnap” was developed to standardize diabetic foot images (52). Through assessments of various scenarios presented by different practitioners, a trial involving 60 patients was conducted to test the stability of FootSnap. The Jaccard similarity index (JSI) was used to confirm the reproducibility of the foot images. The resulting JSI values for diabetic feet were 0.89–0.91, with values of 0.93–0.94 for the control group, which suggested that the app had excellent reliability. Kaabouch et al. (53) used asymmetry analysis in conjunction with a genetic algorithm to analyze thermal images and thereby facilitate the early detection of foot ulcerations and the assessment of skin integrity. This study found that the tested technology was a feasible means of detecting inflammation and could effectively predict potential foot ulcerations.

Katigari et al. (54) developed an expert system based on fuzzy logic and applied it to the medical records of 244 patients diagnosed with diabetic neuropathies; they found that the system could intelligently determine the severity of diabetic neuropathies with a sensitivity of 89%, a specificity of 98%, and an accuracy of 93%.

With the development of information systems and technologies, the digital data collected from diabetes patients is growing exponentially. From the above observations, it is clear that AI technologies employing complex and precise methods can serve as useful management tools in processing diabetes databases to derive valuable information. Accordingly, AI plays a key role in these relevant systems as an auxiliary routine management tool for diabetes patients.

## Conclusions

We have summarized that the most representative applications of artificial intelligence in diabetes education and management (Table 1). As described above, the diabetes education and management is an essential means of improving the quality of disease management. As a consequence, the integration of education and management approaches with mobile health and AI technologies has become an unstoppable trend. However, we've noticed that there are cons and pros of the wide range of AI-based methods. However, the applicability of one algorithm is problem and data specific. For example, in diabetes classification analysis, based on the classification criteria and characteristic distribution of data, some instances may generate good results with directly applying standard methods supplied by common data analysis tools. However, there are also situations where more advanced models are to be developed to infer a clearer layout of the analyzing object. We will proceed with discussing and comparing the strengths and drawbacks of some well-known AI-based methods when certain standards and benchmark data sets are prepared in our future work.

**Table 1:** Examples of the most representative publications on AI applied to diabetes education.

**Method**	**Application**	**Description**	**References**
SVM ML	Diabetes prediction	SVM are recently one of the most popular and flexible ML algorithms used for classification, they are being used to discover valuable knowledge from large databases such as to test the feasibility of using data collected in electronic medical records for development of effective models for diabetes risk forecasting. But the approach is tolerant to a reasonable number of false positives.	(24, 25)
ANN DL	Diet Guidance Retinopathy assessment Exercise guidance	The most widely used techniques are artificial neural networks (ANNs). ANN are based on interconnected neurons, that means, the human brain function. A deep learning algorithm (DL), can be considered and evolution of ANN. For example, based on the techniques of ANN, then proposed a regression model that could be used to automatically analyze the exercise levels of patients wearing accelerometers and heart monitors and monitor changes in glucose levels that occurred while the subjects were exercising.	(28, 30, 32, 33) (50, 51) (34)
CBR	Insulin dose recommendation	Case-based reasoning (CBR) is used to calculate an individualized insulin bolus using an insulin intravenous bolus calculator, thereby achieving optimal glucose levels in patients and optimizing insulin treatment. CBR learns from experiences of past similar meals, which are described in cases through a set of parameters (e.g., time of meal, exercise). However, there are some limitations of CBR, as its application needs to get a large sample size and is often excessively time-consuming.	(39, 41)
GA	Blood sugar monitoring Foot ulcer prediction	GA simulates natural selection by creating a population of individuals (solutions) for optimization problems. Recently this technique is applied on the early detection of foot ulcers. This methodology involves three steps: segmentation, geometric transformation, and asymmetry analysis.	(43, 44) (52, 53)
FL ES	Hypoglycemia detection Peripheral neuropathy	ES are defined as systems with the ability to capture expert knowledge and facts. Fuzzy systems are one of the most common ES used in the field of diabetes. It is a new version of expert systems that uses fuzzy logic for data processing and a self-adaptive learning algorithm.	(40, 45) (54)
DT	Diabetes management	DT is most often created based on a learning algorithm. By recording information on diet, exercise, pharmaceutical use, and blood sugar levels, the application of DL in the systems can combine patient- and physician-support tools for the purpose of improving disease outcomes.	(46, 48, 49)

In conclusion, the use of AI in educational interventions, while promising, faces certain challenges. In spite of the fact that research on the use of digital and intelligent tools in diabetes management is expanding rapidly, most relevant studies lack sufficient numbers of samples or fail to determine whether the results of the tested intervention have clinical significance. To fully and effectively realize AI-based models and establish individualized educational intervention strategies for patients with different needs, it is necessary to achieve the following: (1) gather large amounts of patient data and establish individualized patient data profiles, (2) establish in-depth crossover between the professional knowledge of physicians and AI technologies, (3) continuously update and enlarge the existing knowledge bases, (4) perform standardized, randomized control studies involving clinical practice, and (5) involve both clinicians and patients jointly in a system designed to optimize effectiveness. The resolution of these challenges will depend on scientific research, regulation, and standardization of the targeted areas. In addition, other current challenges include technological, philosophical, and ethical dilemmas, as well as issues surrounding user data security and privacy, and even legal hurdles. Among these issues, information security is a new challenge for the AI era. Further efforts will be needed to promote the rapid and effective application of AI to medical fields.

Despite these challenges, the medical applications of AI have been developing at an extremely rapid rate, and the prospects and research value of these applications cannot be denied. The future use of integrated and comprehensive applications of individual AI technologies in diabetes education can provide full-course, individualized, and intelligent education and thereby provide patients with a lifetime of guidance and protection.

## Author Contributions

JL wrote the manuscript. JH contributed to the definition section. XL provided ideas. LZ polished the article.

## Conflict of Interest

The authors declare that the research was conducted in the absence of any commercial or financial relationships that could be construed as a potential conflict of interest.

## Abbreviations

AI: artificial intelligence
ANN: artificial neural network
CBR: case-based reasoning
ES: expert systems
FL: fuzzy logic
GA: genetic algorithms
ML: machine learning
RBR: rule-based reasoning
SVM: support vector machines.
